# Case Reports and Artificial Intelligence Challenges on Squamous Cell Carcinoma Developed on Chronic Radiodermitis

**DOI:** 10.3390/jcm14113921

**Published:** 2025-06-03

**Authors:** Gyula László Fekete, Laszlo Barna Iantovics, Júlia Edit Fekete, László Fekete

**Affiliations:** 1 Department of Dermatology, George Emil Palade University of Medicine, Pharmacy, Science and Technology of Targu Mures, 540142 Targu Mures, Romania; 2 CMI Dermamed Private Medical Office, 540530 Targu Mures, Romania; 3 Department of Electrical Engineering and Information Technology, George Emil Palade University of Medicine, Pharmacy, Science and Technology of Targu Mures, 540142 Targu Mures, Romania; 4 National Institute of Public Health, Regional Center for Public Health, 540142 Targu Mures, Romania; 5 Doctoral School, George Emil Palade University of Medicine, Pharmacy, Science and Technology of Targu Mures, 540142 Targu Mures, Romania

**Keywords:** radiodermitis, radiotherapy, squamous cell carcinoma, machine learning, deep learning, clinical decision support system, artificial intelligence, inflammatory skin processes, dystrophic skin processes, ionizing radiation, mammary adenocarcinoma, Chaoul rays, irradiation of the skin, skin injury, intelligent agent-based system

## Abstract

**Background/Objectives:** Radiodermitis is an inflammatory or dystrophic skin process caused by the direct action of ionizing radiation. The primary objective was to study two clinical cases. The secondary objective was to propose the foundations of an intelligent system for decision support in complex cases of radiodermitis diagnosis that can operate even in the case of a low amount of available clinical data that can be used for training. **Methods:** The first case is a female patient, aged 74 years, with squamous cell carcinoma on a chronic radiodermitis site, which appeared after 20 years of local radiotherapy treatment for mammary adenocarcinoma. Dermatologic examination revealed five round-oval nodules between 2 and 8 cm in diameter. They were pink colored with lilac edges, hard and infiltrated on palpation, adherent to the subcutaneous tissue, painless, and located above and lateral on the right chest and the upper region of the right hypochondrium. The second case concerns a 60-year-old patient with verrucous squamous cell carcinoma appearing on a chronic radiodermatitis 40 years after local radio-therapeutic treatment with Chaoul rays for a deep right temporal region mycosis. There are presented artificial intelligence (AI) challenges regarding the application of advanced hybrid models in decision support for diagnosis of difficult radiodermitis cases, in that intelligent computing must be made in the context of very little available data, and collaboration between physicians is necessary. **Results:** Both cases were confirmed by histology as squamos cell carcinomas. In the AI research, the adaptation of the IntMediSys intelligent system was proposed for solving complex cases of radiodermitis. The proposal integrates different AI technologies, which include agents, intelligent computing, and blackboard systems. **Conclusions:** The presented first cases confirm the presence of a squamous cell carcinoma that appeared on chronic radiodermitis after a long latency. The foundations of a highly complex collaboration and decision support system that can assist physicians in the radiodermitis diagnostics establishment that opens the path for further development are presented.

## 1. Introduction

Radiodermatitis is an inflammatory or dystrophic skin process produced by the direct action of ionizing radiation. Stevens, in 1896 [1], was the first to report on the appearance of skin lesions caused by X-rays. Later in 1901, Becquerel and Curie observed the appearance of erythema and ulcerations on skin accidentally or voluntarily exposed to radiation. According to various authors, it is accepted that chronic radiodermatitis undergoes an epitheliomatous transformation in 10–34% of cases. Both squamous and basal cell epitheliomas can develop. The latency period is between 4 and 42 years. For squamous cell epitheliomas, the latency period is shorter (3–25 years) than for basal cell epitheliomas (31–35 years) [1]. In the case of post-therapeutic radiodermatitis, the latency period is shorter than it is in the professional one [2]. Most of the carcinomas that appear are squamous cell carcinomas, and develop metastases in 20% of cases [3].

Skin irradiation can lead to complex tissue injury and the attraction of inflammatory cells, leading to the destruction of cells from the basal epidermis, endothelial, and vascular cells, and the reduction of the number of Langerhans cells [4]. Radiation-induced keratinocyte destruction produces Deoxyribonucleic acid (DNA) remodeling insult via the p53 activation pathway and the simultaneous release of inflammatory cytokines as a consequence of free radical generation [5]. The main cytokines involved in this reaction are interleukin 1 and 6, TNF-α, and TGF-β [6]. It has been shown that irradiation induces a significant alteration of the p53 protein, a fact that may be responsible for the change in the epithelial maturation process and the increased risk of developing carcinomas due to radiodermatitis [5]. At the same time, the presence of keratinocytes testifies to the increased expression of the epidermal growth factor receptor (EGFR), possibly as a mechanism for the repopulation of the irradiated areas [7]. Two types of radiodermatitis caused by ionizing radiation are classically recognized: early or acute forms and late or chronic forms. The former appears shortly after radiation exposure, usually in the first 6 months. The chronic ones appear after a longer period, and/or subsequent irradiation [4]. Skin lesions from chronic radiodermatitis can be the prolongation of the symptoms of acute degree III radiodermatitis. They can appear in areas where there was radiodermatitis of degree I or II or in areas where there was no acute radiodermatitis, but which were previously treated with X-rays or have been subjected for a long time to repeated irradiation with low doses of radiation [8]. Several forms of chronic radiodermatitis are described, the most common of which are the atrophic and ulcerative forms. The atrophic form of the presented case is a chronic form where the skin in the affected area is atrophic, thin, and shiny, matte white or pink, sometimes with depigmented areas alternating with hyperchromic areas. All of this atrophic area is sprinkled with numerous telangiectasias, and pilosity is absent [9]. Chronic radiodermatitis has several drawbacks: it is unsightly, persists indefinitely, gets progressively worse, and can undergo malignant transformation [4]. Squamous cell carcinomas appearing on radiodermatitis are predominantly ulcerative, ulcero-vegetative, and less commonly verrucous, as in the presented case [10]. The remainder of the paper is organized as follows. In the next section, we review several representative Artificial Intelligence (AI) models that could be further adapted and extended for decision support in squamous cell carcinoma, developed based on chronic radiodermitis. These could prove capable of performing a wide variety of processing and analysis in different stages of clinical care and diagnosis, starting from prevention to the elaboration of diagnostics. Even if some of the AI methods are applied to internal organs, they can be adapted and applied to skin diseases. Section 3 presents the two clinical cases, which also describe challenges for AI. In Section 4, the discussions related to the studied clinical cases and our proposal for the development of a highly intelligent decision and collaboration support system for physicians are presented. In the last section, the main conclusions of the research are outlined.

## 2. Survey on Artificial Intelligence Applications in Decision Support

In this section, some relevant AI developments that stand out as the basis of our proposal for the AI system are presented. Risk factors of a disease, if known, can have a preventive effect on the occurrence of the disease. However, the identification of risk factors is very important. The study of various background risk stratification of cutaneous squamous cell carcinoma SCC (cSCC) is very important in various clinical decision-making processes [11].

Most SCCs have a favorable prognosis, with only about 2% of SCC cases being fatal [12]. Early detection and treatment of SCCs leads to a cure rate of 95%. In this context, AI with machine learning (ML) and often deep learning (DL) models could provide significant decision support by predicting diagnosis, prognosis, and survival analysis. Such support could be useful for residents and even for experienced physicians.

A compelling example would be the situation in which a clinical decision support system (CDSS) helps a primary care physician, who is not a dermatology specialist, to diagnose skin diseases. Such clinical decision support systems can use heterogeneous data sources, such as laboratory analyses and images, and can even combine artificial intelligence-based reasoning with human medical reasoning [13]. These decision support systems will only assist physicians in their decision making, they will not replace them.

### 2.1. AI Methods Applied for the Analysis of External Organs to Provide Decision Support

The research [14] conducted in 2019 presents a study on different ML classification models, 2-class decision forest, 2-class decision jungle, 2-class logistic regression, and 2-class neural network’s ability to predict oral squamous cell carcinoma (OSCC). The cohort studied consisted of over 30,000 patients. The model used for prediction was based on a variety of sources, such as patients, tumors, treatment institutions, and treatment types. The results obtained with the most robust classifier, the decision tree, showed that it predicted 5-year overall survival with the following performance metrics: precision of 71%, accuracy of 71%, and recall of 68%, with an Area Under the Curve (AUC) of 0.8, 95% Confidence Interval (CI), 0.79–0.81. The 2-class decision forest was less accurate with an AUC of 0.68, 95% CI, 0.67–0.7, accuracy of 65%, precision of 69%, and recall of 52%. Challenges identified consisted of aspects related to some machine learning models and their effective clinical implementation.

The 2021 study [15] presents an in-depth review of ML-based diagnosis and prognosis of OSCC. It showed the limitations of physicians in implementing machine learning-based models, which can be used in daily clinical practice. The study included very important databases such as Scopus, Medline, Pub-Med, Web of Science, and the Institute of Electrical and Electronics Engineers (IEEE) from the beginning to February 2020. Of the 41 studies identified by interest, most of the models consisted of support vector machine (SVM) and different types of artificial neural networks (ANN). The specificity of the models ranged from 0.57 to 1.00, the sensitivity from 0.70 to 1.00, and the accuracy from 63.4% to 100%.

cSCC has metastatic potential and may cause mortality. Based on this issue, clinical measurement of metastatic risk is very important. The study conducted [16] in 2022 presented an AI algorithm, called residual neural network, specialized in the detection of metastatic primary cSCCs. The obtained results show an AI algorithm capable of recognizing unknown morphological features associated with metastasis. According to [16], the requirements for the algorithm are the prognosis of primary cSCC and the assessment of metastasis risk.

Research [11] conducted in 2023 focused on investigating how machine learning models could help stratify patients with cSCC by risk based on various clinical and histopathological factors. The research was conducted on a retrospective cohort of 104 patients with cSCC, examining clinical and histopathologic risk factors. Cellular morphometric biomarkers (CMBs) were detected based on ML models, which were used to derive the so-called cellular morphometric risk score (CMRS), based on which cSCCs can be classified with different prognoses. The main conclusion of the study was that CMRS is useful for cSCC risk stratification beyond classical clinical and histopathological risk features.

Research [17] conducted in 2024 applied logistic regression (LR), which is a supervised ML model, for the identification and delineation of SCC. In the present study, the MALDI-MSI database was labeled based on histology. The results outline the performance of ML using MALDI-MSI data to delineate and characterize SCC. The obtained results suggest a promising direction for the development of mass spectrometry methods in the clinical diagnosis of SCC. The research presented in [18] presents the assumptions that must be met by binomial logistic regression so that the application is correct. Frequently in medical research, they are not considered assumptions that the data should pass so that the models’ applications are correct.

### 2.2. AI Methods Applied to Internal Organs to Provide Decision Support

The study presented in [19], conducted in 2023, focused on the survival prognosis of esophageal squamous cell carcinoma (ESCC). The main goal of the research was to increase the accuracy of individualized patient dosing regimens. Several ML methods were investigated. These included Recursive Partitioning and Regression Trees (Rpart), Elastic Net Regularized Generalized Linear Models (Elastic Net), Gradient Boosted Machine (GBM), Random Survival Forest (randomForestSRC), Gradient Boosting with Component-wise Linear Models (GLMboost), and machine learning techniques extended Cox Proportional Hazards (CoxPH) were all used to fit models predicting survival outcomes. The models were trained on a dataset of 1954 ESCC patients using 27 clinical characteristics validated on a dataset of 487 ESCC patients. Elastic Net, Random Forest, and the machine learning-enhanced CoxPH model showed comparable performance in predicting mortality risk in ESCC patients. They outperformed GBM, GLMboost, and Rpart. The risk scores obtained using the CoxPH model classified ESCC patients into low-, intermediate-, and high-risk groups with different 3-year overall survival probabilities. The general CoxPH model was found to be sufficiently good for interpretive studies.

The motivation of the study [20] performed in 2023 consisted of the fact that patients with lung squamous cell carcinoma (LUSC), unfortunately, are often diagnosed only at an advanced stage and as a result may have a poor prognosis. In this context, the identification of novel biomarkers for LUSC may be very useful. In the performed study, various datasets from the NCBI-GEO repository were included. From these data, a dataset containing known cancer driver genes was selected. ML classifiers were used to select the best features. Differential gene expression analysis was also performed. In addition, survival and enrichment analyses were performed. Among the ML models investigated, the k-Nearest Neighbors (kNN) classifier performed best. Lasso penalized Cox regression further reduced the number of genes investigated. The study showed that ML can successfully identify biomarkers for LUSC.

The research presented in [21], carried out in 2023, focused on reducing the limitation of current clinical decision support systems specialized in cutaneous squamous cell carcinoma (cSCC), which do not provide estimates of the absolute risk of metastasis, thus reducing the possibility of personalized treatments. They proposed and validated a clinicopathologic model that predicts metastasis in patients with cSCC. The model was able to assign personalized metastasis risk predictions to patients with cSCC based on patient-specific risk factors and reported histological data. It enables physicians and clinical decision support systems to identify patients with high-risk cSCC. It is also capable of providing personalized treatment.

### 2.3. Concluding Remarks Regarding Research Carried Out Worldwide

This section presented an overview of the conducted bibliographic study on various models that can be applied to different aspects related to the processing of various data, diagnosis, and prognosis of squamous cell carcinoma, including its evolution, which we reported on chronic radiodermitis, opening the path of future studies and research.

## 3. Cases Presentations

In this section, we report the two cases.

### 3.1. Case of a 74-Year-Old Female Patient

**Figure 1:**
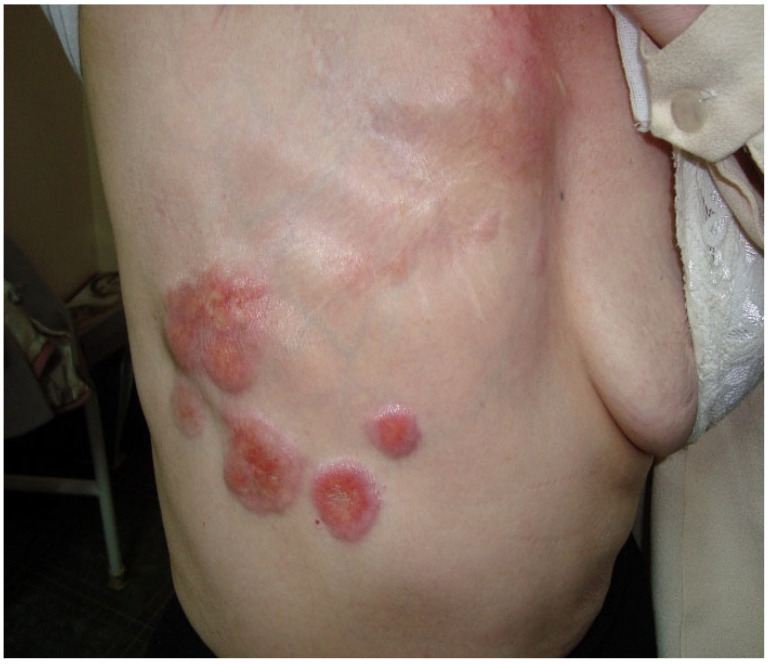
Clinical appearance of carcinomatous lesions.

We present the case of a 74-year-old female patient with squamous cell carcinoma (SCC) that appeared 20 years after local radiotherapy treatment for mammary adenocarcinoma. The patient was diagnosed in 1990 with right breast adenocarcinoma in clinical stage III A T2N2M0, for which total mastectomy with removal of the axillary nodes was performed. Afterward, systemic treatment with cytostatics and local radiotherapy in a total dose of 40 Gray followed. She underwent radiotherapy treatment of 2 Gray per daily session, 5 days a week for 4 weeks, receiving 40 Gray doses of radiotherapy. Figure 1 presents the clinical appearance of carcinomatous lesions. During the dermatological examination, we observed five round-oval lesions, with a diameter between 2 and 8 cm, pink in color with a lilac outline, hard and infiltrated to the touch, adherent to the subcutaneous tissue, and painless, located at the level of the anterior and lateral chest, as well as in the upper part of the right hypochondrium.

**Figure 2:**
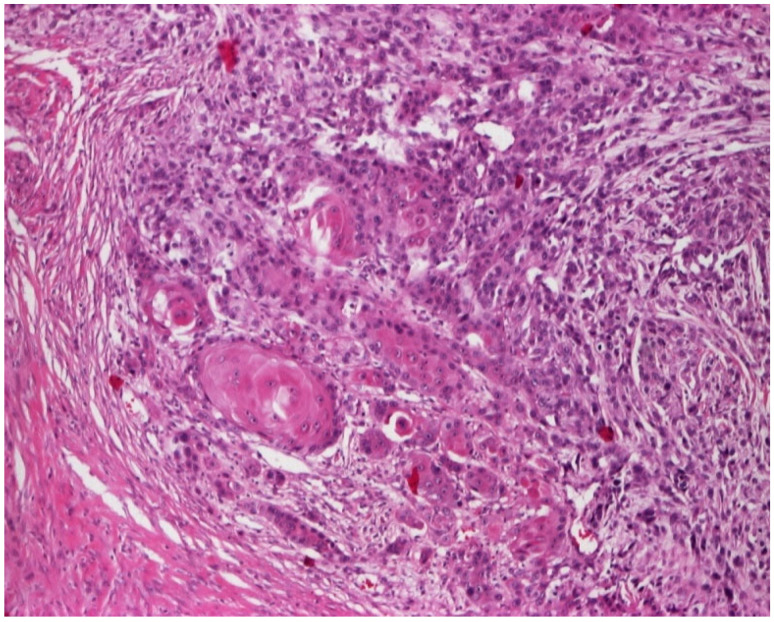
Squamous cell carcinoma—histological aspect.

These nodules developed over approximately 5 months, with the first nodule appearing as the largest. In addition to the previously described lesions, a dark red, brick-colored atrophy-scar plaque can be detected on the right parasternal region. From the anamnesis, it appears that the area of this plaque and the area of the largest nodular formation of cc 8 cm in diameter were the areas of radiotherapy application 20 years ago. These two atrophy-scarring areas have persisted for about 15 years, and we can consider them chronic radiodermatitis lesions. No palpable adenopathies were found during the general clinical examination. The laboratory analyses carried out were of no pathological importance. Imaging investigations: lung X-ray, abdominal ultrasound, and computed tomography did not detect distant metastases. Figure 2 presents the histological aspect of squamous cell carcinoma. The histopathological examination performed on the largest tumor lesion confirmed the diagnosis of squamous cell carcinoma, and the one performed on the right parasternal plaque confirmed the diagnosis of chronic radiodermatitis, finding an alternation of epidermal atrophy, hyperacanthosis, and hyperkeratosis. In the dermis were found endovasculitis with dilatations of small vessels, true angiomatous lakes, such as the absence of hair follicles, sebaceous, and sweat glands. The treatment recommended to the patient was surgical.

*Case 1-AI-Suggested AI Further Development*

A 2022 reference study [22] discussed advanced AI-based methods for image recognition in dermatology. It demonstrates that 3D image models can be used for decision support by screening and labeling pigmented skin lesions. This can help with the documentation of lesion sites. Aspects such as opportunities and emerging risks of AI applications in dermatology are discussed. At the same time, future trends and perspectives in the important field of image-based AI in dermatology are summarized.

A future undeveloped ML/DL application is the automatic detection and diagnosis of carcinomatous lesions on the skin, as shown in Figure 1, which can be easily obtained even with a simple camera. This can be achieved by using ML/DL-based medical imaging techniques, which must also be explainable. Such assistance could be especially helpful for less experienced clinicians, general practitioners, and residents. For machine learning algorithms to be applicable, large collections of images with carcinomatous lesions are required. One of the challenges in this sense is the limitation of available images in the learning processes, which could require specialized techniques to generate variants of images to increase the number of images used to train the models. Another difficulty could be to provide the ML/DL algorithms with explainability, which could allow for more accurate decisions by the clinicians and even additional descriptive characterizations, such as the severity of the disease, measure of the affected surface diameter, positions of the legions, and color and outline of the legions. Another interesting open research direction is the development of AI algorithms for the prognosis evolution of skin lesions and the disease.

### 3.2. Case of a 60-Year-Old Male Patient

**Figure 3:**
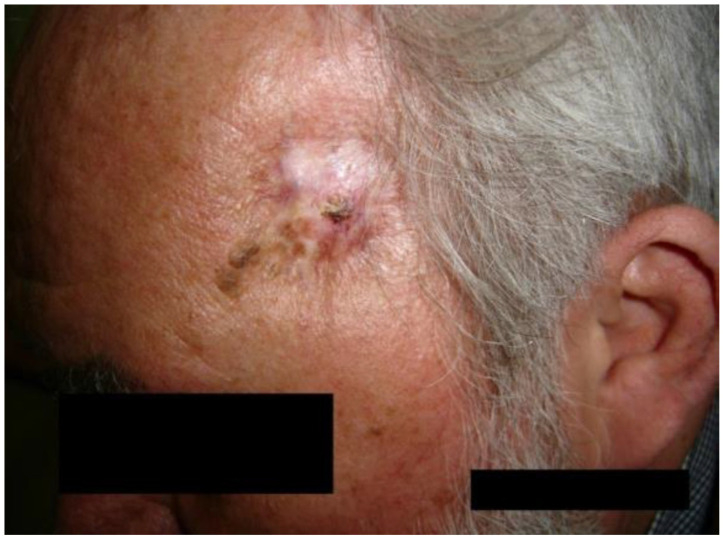
Verrucous squamous cell carcinoma appearing on chronic radiodermatitis—clinical aspect.

We present the case of a 60-year-old male patient with verrucous squamous cell carcinoma appearing on chronic radiodermatitis 40 years after local radio-therapeutic treatment with Chaoul rays for a deep right temporal region mycosis. He underwent radiotherapy treatment of 200 rad per daily session, 5 days a week for 2 weeks, receiving a total of 2000 rad radiotherapy doses, equivalent to 20 Gray doses. During the dermatological examination, a formation with a diameter of 1 cm was observed, with a verrucous surface, and an adherent gray color. It was painless and located at the level of the right temporal region on the surface of a pink atrophic-cicatricial plaque, with telangiectasias on the surface (Figure 3).

No palpable adenopathies were found during the general clinical examination. The laboratory analyses performed were without pathological significance. Imaging investigations did not detect distant metastases.

**Figure 4:**
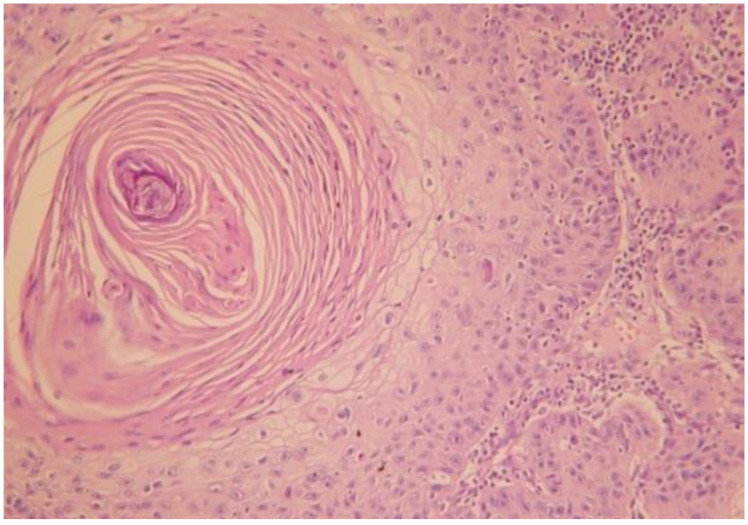
Well-differentiated verrucous squamous cell carcinoma with parakeratotic globule (HE 20× stain).

**Figure 5:**
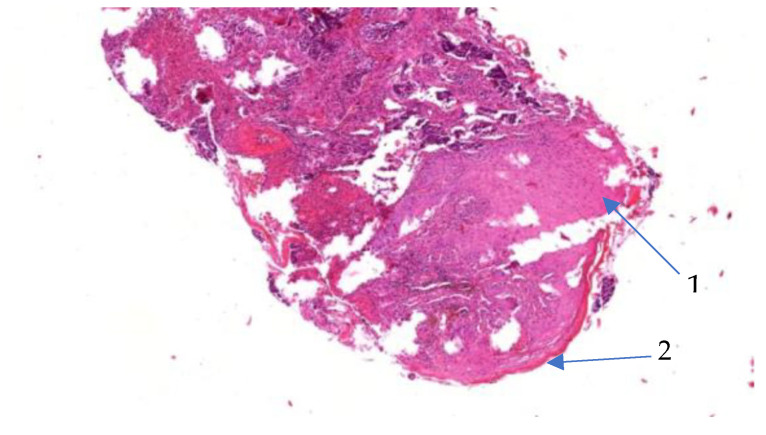
Histological appearance of chronic radiodermatitis (10× HE stain), for patient no. 1.

**Figure 6:**
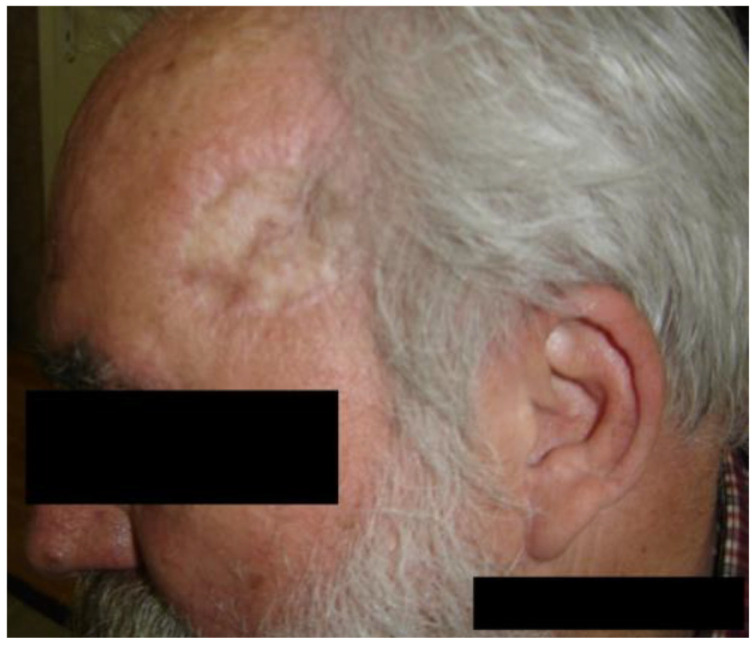
Clinical appearance one month after surgical treatment.

The histopathological examination confirmed the diagnosis of well-differentiated verrucous squamous cell carcinoma with minimal cytonuclear pleomorphism and formation of parakeratotic globules, as well as chronic radiodermatitis lesions with hyperkeratotic epidermis. The epidermal cells showed a discrete disorganization, with dyskeratotic lesions and rare nucleo-cytoplasmic atypia. The elastic fibers were fragmented and destroyed (Figure 4 and Figure 5). The arrows used in Figure 5 indicate the histological description as hyperacanthosis (arrow with number 1) and hyperkeratosis (arrow with number 2). Figure 6 shows the clinical appearance one month after surgical treatment of the 60 years old male patience.

The recommended and performed treatment was surgical with the extirpation of the tumor and the radiodermatitis area, applying an autologous graft (Figure 3).

*Case 2-AI—Suggested AI Future Development*

This case shows an important AI/ML-based imaging problem that can be approached in research, more concretely, the detection of verrucous surface on the skin (like the example presented in Figure 3), which can be located even on the face and have different characteristics such as color, localization, and surface dimension. Challenges could be the small number of available images that can be used by the AI/ML algorithms, their enrichment with explainability, and the ability to make predictions regarding the evolution.

## 4. Discussion

SCC is a type of malignant tumor that develops from the keratinizing cells of the epidermis or its appendages and is the second most prevalent form of skin cancer in immunocompetent white individuals and the most common skin cancer in immunosuppressed organ transplantation recipients worldwide [23]. This cancer is locally aggressive and can metastasize, primarily with regional lymph nodes and occasionally to distant organs. Clinically, SCC is often presented as a hardened, hyperkeratotic nodule with central ulceration and a keratin mass or as an ulcerated papule, nodule, or plaque without keratinization. Typically, SCC arises in areas of severe sun damage, often among several nearby actinic keratoses (AKs) [24]. SCC follows the “classic cancer” structure, which includes precursor lesions, tumor progression, and metastasis. The prognosis is determined by tumor-related characteristics such as tumor size, location on the body, depth of invasion into the subcutaneous tissue, perineural involvement, histological differentiation, and the patient’s immune system [25]. The current consensus on cutaneous SCC is that the cancer is caused by a single transformed cell of the keratinocytic lineage and develops as a result of genetic mutations that provide a selective growth advantage. The most prevalent genetic abnormalities detected in SCC are TP53 gene mutations. Dysregulation of p53 pathways appears to be an early event in the carcinogenesis of SCC [26].

Preinvasive lesions or precancerous diseases such as actinic keratoses, cutaneous horns, keratoacanthomas, and Bowen’s disease are the root cause of most SCCs [27,28].

Environmental, phenotypic, and genetic variables all contribute to the likelihood of developing SCC. Ultraviolet (UV) radiation is considered the most significant environmental risk factor [29]. Other significant risk factors for SCC include exposure to physical and chemical carcinogens, immunosuppression, and genetic susceptibility [30,31,32,33].

Ionizing radiation exposure increases the risk of basal cell carcinoma (BCC) and SCC by a factor of three [34]. The risk involved is proportional to the radiation exposure. Larger fractionated doses (>12–15 Gy) are expected to be necessary to promote tumor formation, hence, the risk associated with a particular total dosage may be reduced if a greater number of smaller fractionated doses are administered. Direct DNA damage is the most significant factor. Most ionizing radiation-induced SCCs and BCCs develop decades later, with the majority of tumors developing ~20 years after initial exposure. Secondary tumors are extremely rare in skin locations with significant exposure to high dosages. They are seen in transition zones to clinically normal skin in up to 10% of affected patients. Therefore, including the possibility of “combination” damage (such as cutaneous radiation syndrome in sun-exposed areas) is essential, which significantly modifies the risk assessment.

Before the discovery of efficient systemic antifungal drugs, radiotherapy treatment for tinea capitis was connected to the development of numerous BCCs and SCCs [35].

### AI in Decision Support Squamous Cell Carcinoma Developed on Chronic Radiodermitis

An agent-based system (ABS) can be an individual agent or a cooperative multiagent system. Intelligent agent-based systems represent an emerging field of AI, a feasible solution to highly complex problems in health sciences that include diverse, difficult cases for physicians and health data computing systems, such as comorbidities and very rare cases [36,37]. Their concrete applications are very diverse, ranging from medical imaging [38] to the evaluation of human–AI interactions [39], just to name a few. Although agent technology has a very high potential for intelligent computing, its application in dermatology is rare. We believe that further development of agent-based systems in dermatology is important because agents could integrate and combine a variety of methods and even provide interpretability and explainability of results obtained by applying traditional AI/ML methods. Section 2 presented various applications of machine learning, but one of its fundamental drawbacks was the limitation in interpretability and explainability, which is very important in clinical applications, which, to a large extent, includes the field of dermatology. Agents can cooperate to solve problems more efficiently than other traditional AI technologies, or even cooperate with physicians by interacting at different decision points.

In previous research [13], we presented the foundations of a highly complex cooperative reasoning based on an intelligent multi-agent system and a team of physicians. The proposed so-called Intelligent Medical Hybrid System (IntMediSys), which is an agent-based system, supports the cooperation of teams of physicians and provides intelligent decision support. The proposal is designed to solve very difficult medical diagnosis cases. The proposed innovative collaborative reasoning is an extension of the so-called blackboard-based problem solving proposed in the field of artificial intelligence. Earlier studies [40,41] present the first time the blackboard-based problem solving in AI literature, whose idea originates from the children’s Jigsaw puzzle game, which involves more children who collaboratively construct an image split into parts. Even though it is appropriate for AI-based problem solving for very difficult problems, it has very few implementations due to its emergent high complexity in hybrid problem-solving implementation. Further research [42] provides an in-depth study of the cognitive complexity of designing the human group decision process.

**Figure 7:**
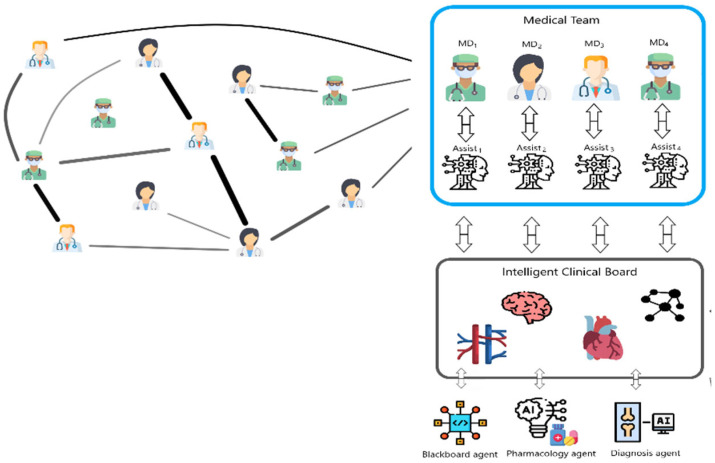
Conceptual representation of the proposed cooperative problem solving.

Figure 7 conceptually shows the main idea of the IntMediSys cooperative solution of a highly complex, rare clinical case by presenting a hypothetical problem-solving scenario. MD_1_, MD_2_, MD_3_, and MD_4_ denote the formed collaborative physicians’ teams that contribute to the problem solving. In [13], the cooperative medical team formation is based on a novel adapted contract net team formation algorithm. The team formation algorithm selects an efficient problem-solving physicians team based on various aspects such as necessary medical specialization, experience, and previous collaborations with other team members. The original contract network technique, which is used as a task assignment protocol to solve the problem of finding the most appropriate agent to perform a particular processing (solve a particular problem), was first introduced in 1980 by Smith [43]. Assit_1_, Assit_2_, Assit_3_, and Assit_4_ represent so-called assistant agents that support physicians during collaborative clinical case solving. Each assistant agent is owned by a physician. In the scenario presented in Figure 1, Assit_1_ is owned by MD_1_, Assit_2_ is owned by MD_2_, Assit_3_ is owned by MD_3_, and Assit_4_ is owned by MD_4_. Assistant agents help physicians by making collaborative problem solving easier and more efficient. For decision support, there are intelligent agents included, called knowledge sources, which can perform different particular types of processing on available data and information during a clinical case resolution to help physicians solve the clinical case. Also, an important aptitude of such implemented agents must be the ability to help physicians offer interpretability and explainability. Such agents must integrate based on the necessary appropriate ML/DL methods, some of them mentioned in Section 2, and extend them with explainability (that is, a limitation of many actual AI developments in dermatology).

Figure 7 presents a scenario in which collaborative problem solving contributions also involve three knowledge source agents called Blackboard agents, Pharmacology agents, and Diagnosis agents. The so-called Intelligent clinical board represents the generic “place” where a clinical case is solved by the joint contribution of physicians, members of the team, assisted by assistant agents, and the knowledge sources. It includes different available data, information, and knowledge that are used during clinical case solving. The scenario represents a complex case in which many types of clinical insights are analyzed.

In conclusion, based on our previous research and bibliographic study, we emphasize the importance of implementing IntMediSys even for solving and researching very rare clinical cases, such as the squamous cell carcinoma developed in chronic rheumatoid arthritis. Difficulties in such research, whose purpose is to generate knowledge, are based on data from very few cases, which could make it impossible to develop traditional machine learning models or other types of processing. Dermatologists also have limitations in formulating relevant conclusions because it is a very rare case based on limited data, information, and knowledge.

IntMediSys could be a feasible solution as it is a collaboration and decision support system for physicians based on specifically designed reasoning. IntMediSys makes physician collaboration easier and more efficient. The integrated multi-agent system is scalable. The agents that provide decision support can be diverse and can be based on a wide variety of artificial intelligence methods, ranging from rule-based systems to natural language processing of clinical manuscripts, and from scientific literature to large language models and transformers. Another strength could be found in the ability to provide advanced interpretability and explainability.

A developed IntMediSys system will be an open system that can integrate different technologies, such as advanced imaging technologies. Case 1-AI and Case 2-AI can be implemented and integrated into individual intelligent cooperative agents, called knowledge sources in our approach, able to help the cooperative medical team in solving rare and complex cases by solving problems such as detection, diagnosis, characterization of medical images, and prognosis regarding the evolution of diseases.

Recent 2024 research [44] shows the promising applications of even generic Large Language Models (LLM) in clinical decision making in dermatology in general and teledermatology in particular. Some aspects of the relatively new LLM are understudied in the literature, such as the ethical, clinical, and practical implications. Aspects such as the effectiveness and challenges of ChatGPT in dermatology are discussed [44], with a focus on clinical applications. IntMediSys could integrate intelligent agents that incorporate technologies based on LLM and transformers, whose applicability in dermatology has already been demonstrated [44].

## 5. Conclusions

The presented cases demonstrate the appearance of squamous cell carcinoma in chronic radiodermatitis after a long latency period. Patients undergoing radiotherapy must be followed to prevent malignant transformation of radiodermatitis. The cure of mammary carcinoma in an advanced stage, as well as the appearance of squamous cell carcinoma on chronic radiodermatitis, are rare clinical entities, which is why we considered it necessary to present this clinical case. The peculiarity of the second case is the development of verrucous squamous cell carcinoma on chronic radiodermatitis, an entity rarely described in the literature.

In addition, the paper presented an overview of machine learning models that could potentially be integrated into a complex clinical decision support system capable of making the diagnosis and prognosis of squamous cell carcinoma developed on chronic radiodermatitis. Finally, it presents the adoption and implementation of a highly complex artificial intelligence-based combined reasoning performed by a so-called Intelligent Medical Hybrid System (IntMediSys) that can be applied for solving very rare, highly complex cases and research as squamous cell carcinoma on chronic radiodermatitis.

## Data Availability

The dataset is available on request from the authors.
